# p21-Activated Kinase 1 (Pak1) Phosphorylates BAD Directly at Serine 111 *In Vitro* and Indirectly through Raf-1 at Serine 112

**DOI:** 10.1371/journal.pone.0027637

**Published:** 2011-11-11

**Authors:** Diana Z. Ye, Shenghao Jin, Ya Zhuo, Jeffrey Field

**Affiliations:** Department of Pharmacology, University of Pennsylvania Perelman School of Medicine, Philadelphia, Pennsylvania, United States of America; Bauer Research Foundation, United States of America

## Abstract

**Background:**

Cell survival depends on the balance between protective and apoptotic signals. When the balance of signals tips towards apoptosis, cells undergo programmed cell death. This balance has profound implications in diseases including cancer. Oncogenes and tumor suppressors are mutated to promote cell survival during tumor development, and many chemotherapeutic drugs kill tumor cells by stimulating apoptosis. BAD is a pro-apoptotic member of the Bcl-2 family of proteins, which can be phosphorylated on numerous sites to modulate binding to Bcl-2 and 14-3-3 proteins and inhibit its pro-apoptotic activities. One of the critical phosphorylation sites is the serine 112 (S112), which can be phosphorylated by several kinases including Pak1.

**Methodology/Principal Findings:**

We mapped the Pak phosphorylation sites by making serine to alanine mutations in BAD and testing them as substrates in *in vitro* kinase assays. We found that the primary phosphorylation site is not S112 but serine 111 (S111), a site that is sometimes found phosphorylated *in vivo*. In transfection assays of HEK293T cells, we showed that Pak1 required Raf-1 to stimulate phosphorylation on S112. Mutating either S111 or S112 to alanine enhanced binding to Bcl-2, but the double mutant S111/112A bound better to Bcl-2. Moreover, BAD phosphorylation at S111 was observed in several other cell lines, and treating one of them with the Pak1 inhibitor 2,2′-Dihydroxy-1,1′-dinaphthyldisulfide (IPA-3) reduced phosphorylation primarily at S112 and to a smaller extent at S111, while Raf inhibitors only reduced phosphorylation at S112.

**Conclusion/Significance:**

Together, these findings demonstrate that Pak1 phosphorylates BAD directly at S111, but phosphorylated S112 through Raf-1. These two sites of BAD serve as redundant regulatory sites for Bcl-2 binding.

## Introduction

Many apoptotic stimuli disrupt the integrity of the mitochondria, causing them to release pro-apoptotic contents such as cytochrome c and apoptosis inducing factor (AIF) [1]. One of the triggers of mitochondrial disruption is the translocation of BAD, a pro-apoptotic member of the Bcl-2 family. BAD activity is regulated by phosphorylation, which reduces its activity [2], [3]. Akt phosphorylates serine 136 (S136); PKA phosphorylates serines 112 (S112) and 155; p90RSK also phosphorylates S112; JNK phosphorylates serine 128 (S128) and threonine 201; Cdc2 phosphorylates S128 [4]–[11].

Raf-1 promotes cell survival through multiple mechanisms. Whereas plasma membrane targeting of Raf-1 activates the classical MEK>ERK (MAPK) cascade but does not protect cells, mitochondrial targeting of Raf-1 protects cells [12]. There are conflicting studies on the role of Raf-1 in BAD phosphorylation [12]–[14], however, the most recent studies using Raf inhibitors suggest that Raf-1 and other Raf isoforms phosphorylate BAD on S112 [15], [16].

Paks (p21-activated kinases), which are the effectors for the small GTPases Rac and Cdc42, have a major role in signaling pathways to promote cell migration, cell proliferation transformation and survival [17]–[22]. Pak1 and other isoforms stimulate phosphorylation at S112 and S136 of BAD *in vitro* and *in vivo*. A survey of 98 signaling antibodies found that phosphorylation at S112 and S136 were pharmacodynamic markers for measuring the effectiveness of Pak inhibitors *in vivo* [23]–[28]. More recent studies have found that Pak stimulation of S112 phosphorylation may be indirect, proceeding through Raf-1 [15], [16]. Additionally, Pak1 and Pak5 stimulate Raf-1 complexes with Bcl-2 and mitochondrial translocation of Raf-1 [15], [29], [30].

Here through *in vitro* mutational analysis, we showed that neither S136 nor S112 of BAD was a significant site of Pak1 phosphorylation. Instead, the vast majority of phosphorylation by Pak1 occurred at S111, a site recently identified in cells. Using transfection assays in HEK293T cells, we also confirmed that Pak1 can phosphorylate S112 through Raf-1. In addition, we found that S111 phosphorylation may precede S112 phosphorylation to facilitate S112 phosphorylation. Bad phosphorylation at S111 was observed in several other cell lines, and inhibition of Pak1 with inhibitor 2,2′-Dihydroxy-1,1′-dinaphthyldisulfide (IPA-3) reduced phosphorylation at S111 and S112.

## Materials and Methods

### Materials

Dulbecco's modified Eagle's medium (DMEM) and fetal bovine serum were from Invitrogen (Carlsbad, CA). FuGENE 6 transfection reagent, complete protease inhibitor cocktail tablets were from Roche (Indianapolis, IN). Rabbit polyclonal antibodies against BAD, phospho-Ser112, phospho-Ser136, ERK, phospho-ERK, phospho-c-Raf (Raf-1) (Ser338) were from Cell Signaling Technology (Beverly, MA). Antibodies against Bcl-2 (N-19) and 14-3-3 (C-16) were purchased from Santa Cruz Biotechnology (Santa Cruz, CA). 5-Iodo-3-[(3,5-dibromo-4-hydroxyphenyl)methylene]-2-indolinone (GW5074), rapamycin, and 2,2′-Dihydroxy-1,1′-dinaphthyldisulfide (IPA-3) were from CalBiochem (La Jolia, CA). PD098059 and H89 were purchased from Sigma (Saint Louis, MO). Glutathione SepharoseTM 4B was purchased from Amersham Pharmacia Biotech (Uppsala, Sweden).

### Plasmids

Plasmids expressing a Myc-tagged wild-type (WT), a kinase-dead (KD or K299R) and a kinase-activated (T423E) version of Pak1 cloned into the pCMV6 vector have been described elsewhere [31]. The plasmids used to generate GST-BAD (aa104-141) fusion proteins were provided by the late Dr. Stanley Korsmeyer. Fragment GST-BAD wild-type (WT), GST-BAD S111A, GST-BAD S112A, GST-BAD S136A, GST-BAD S112/111A, GST-BAD S111/136A, GST-BAD S112/136A, GST-BAD S112/136/108A, GST-BAD S112/136/134A, GST-BAD S112/136/111A, and GST-BAD S112/136/128A were all cloned into the pGEX-4T-1. pBAC-his-hPak1, which expresses a human Pak1 cloned into a baculovirus expression system, was provided by Dr. Jonathan Chernoff. pEBG-BAD encoding GST tagged with the full length BAD was purchased from Cell Signaling. This plasmid was used to create GST-BAD S111A, GST-BAD S112A, GST-BAD S136A, GST-BAD S111/112A, GST-BAD S111/136A, GST-BAD S112/136A, and GST-BAD S111/112/136A by mutating the specific amino acid(s) within the full length BAD. Plasmids expressing mutant BAD (full length or aa104–141) were constructed using site-directed mutagenesis (Stratagene).

### Antibody Production

The S111-phosphorylated peptide, ETRSRH[pS]SYPAGTE, corresponding to amino acid residues 105 to 118 of mouse BAD, was synthesized (ResGen, Invitrogen Corporation), conjugated to KLH and injected into rabbits for antibody production. To affinity purify the antibody, the peptide was cross-linked to NHS (N-hydroxysuccinimide)-activated Sepharose 4 Fast Flow beads (Amersham-Pharmacia Biotech) as instructed by the manufacturer and the beads were used to purify the anti-phospho-BAD (S111) serum.

### Cell Culture and Transfection

HEK293T, and a malignant peripheral nerve sheath tumor (MPNST) cell line ST88-14, were grown at 37°C in 5% CO_2_ and cultured in DMEM containing 10% fetal bovine serum, penicillin (100 units/ml), and streptomycin (100 µg/ml). Cells were transfected using FuGENE 6 for 24 hr, and then starved overnight before harvesting. A human lung cancer cell line H358, and MPNST cell lines, 90-8 and STS26, were cultured in RPMI containing 10% fetal bovine serum, penicillin (100 units/ml), and streptomycin (100 µg/ml).

### Immunoblot and Immunoprecipitation

Cells were transfected with appropriate vectors. Cell extracts were prepared using lysis buffer (50 mM HEPES pH 7.5, 0.15 N NaCl, 1.0% NP-40, 1 mM EDTA, 1 mM EGTA, 1 mM glycerophosphate, 0.5 mM vanadate and 10% glycerol) supplemented with 1 mM phenylmethlsulfonyl fluoride and the recommended concentration of Complete protease inhibitors (Roche). Protein concentration was assessed using the Bio-Rad D_c_ Protein Assay Kit and equal amounts of proteins were separated by SDS-PAGE, transferred to polyvinylidene difluoride membranes (Immobilion P; Millipore Corp). Blots were blocked for 1 hr in TBS-T (Tris-Buffered Saline with Tween-20) buffer supplemented with 5% nonfat milk, and incubated with primary antibodies overnight at 4°C. After washing, blots were incubated with secondary antibodies, and then detected using the enhanced chemiluminescence detection system (Amershan Pharmacia Biotech). The immunoprecipitation assay was carried out as following: whole cell lysates containing the same amount of total protein were incubated with glutathione beads for 4 hr at 4°C. Beads were collected by centrifugation and washed twice with the lysis buffer and once with 1xPBS. Proteins were eluted by boiling in 1xSDS sample buffer, and subjected to immunoblotting, and then probed with antibodies against p-Ser112 BAD, p-Ser111 BAD, Bcl-2 or 14-3-3.

### Kinase Assays

Protein kinase assays were conducted by incubating a mixture of proteins with the indicated substrates in 1X kinase buffer (10 mM MgCl_2_, 40 mM Hepes, pH 7.4) supplemented with 5 µM ATP or where indicated 5 µCi of γ- [^32^P] ATP for 30 min at 30°C in a reaction volume of 25 µL. The reaction was terminated with 1XSDS sample buffer, followed by SDS-PAGE, autoradiography or Western blotting.

For peptide kinase assays, peptides were dissolved in Tris/EDTA buffer to make a stock concentration of 20 µg/µL. The Ninhydrin test was run with 2 µL of each sample spotted on a Flexible Thin layer Chromatography (TLC) plate to ensure the equal loading of the peptides. The peptides were incubated with Pak and Cdc42^L61^ with or without Raf-1 in 1X kinase buffer (10 mM MgCl_2_, 40 mM Hepes, pH 7.4) supplemented with 20 µM ATP and 1 µCi of γ- [^32^P] ATP for 30 min at 30°C in a reaction volume of 28 µL. The reaction was terminated with 1X Tricine SDS sample buffer (Invitrogen), run on a an SDS-PAGE tricine gel (Invitrogen) and analyzed by autoradiography.

**Figure 1:**
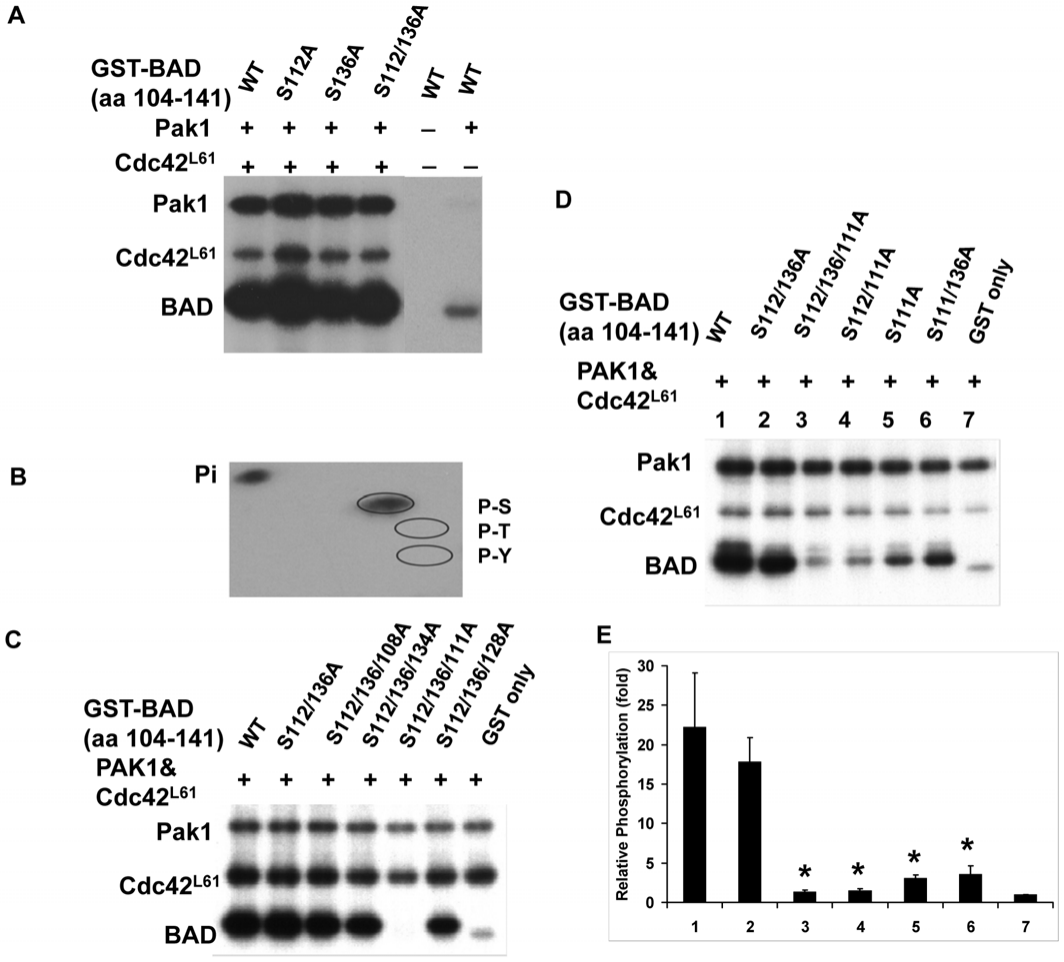
Pak1 phosphorylates BAD (aa 104–141) primarily on S111 instead of S112/136. (A) Pak1 phosphorylates S112/136A double mutant of BAD. 4 µg of each GST fusion protein of fragment BAD wild type (WT aa 104–141) or mutant BAD (aa 104–141) with the mutation of serine-to-alanine at 112, 136 or both sites was used as substrate for *in vitro* kinase assay with Pak1 in the present or absence of Cdc42^L61^, which was used to activate Pak1. Kinase assays were carried out using γ- [^32^P] ATP for 30 min at 30°C as described in Materials and Methods. One quarter of each reaction was separated by 10% SDS-PAGE, and phosphorylated proteins were detected by autoradiography. The position of phosphorylated Pak1, Cdc42^ L61^, and BAD WT and mutants were determined according to their sizes as shown in the Figure S1C. (B) Phosphoamino acid analysis of phosphorylated GST-BAD (aa 104–141). GST-WT BAD (aa 104-141) was phosphorylated by Pak1, and the proteins were run through SDS-PAGE. The phosphorylated BAD was isolated and subjected to acid hydrolysis and analyzed on a TLC plate as described in Materials and Methods. P-S, P-T and P-Y represent phosphor-serine, phosphor-threonine and phosphor-tyrosine, respectively. Pi indicates orthophosphate. (C–E) Mutation at S111 abolished BAD phosphorylation by Pak1. Kinase assay was carried out as in Figure 1A, except that substrates were the indicated GST-BAD mutants (aa 104-141). Panel E shows phosphorimager quantification of the bands in panel D (n = 3).

For autoradiography, the phosphorylation gel was exposed to a phosphor screen and the phosphorylated BAD bands were quantified using ImageQuant V1.2 software. The “Relative Phosphorylation” was calculated based on the level of phosphorylation in GST alone for Figure 1E or lane 10 for supplemental figure 2B, which was set to a value of “1”. Statistical analysis was performed using student t-test for significance.

**Figure 2:**
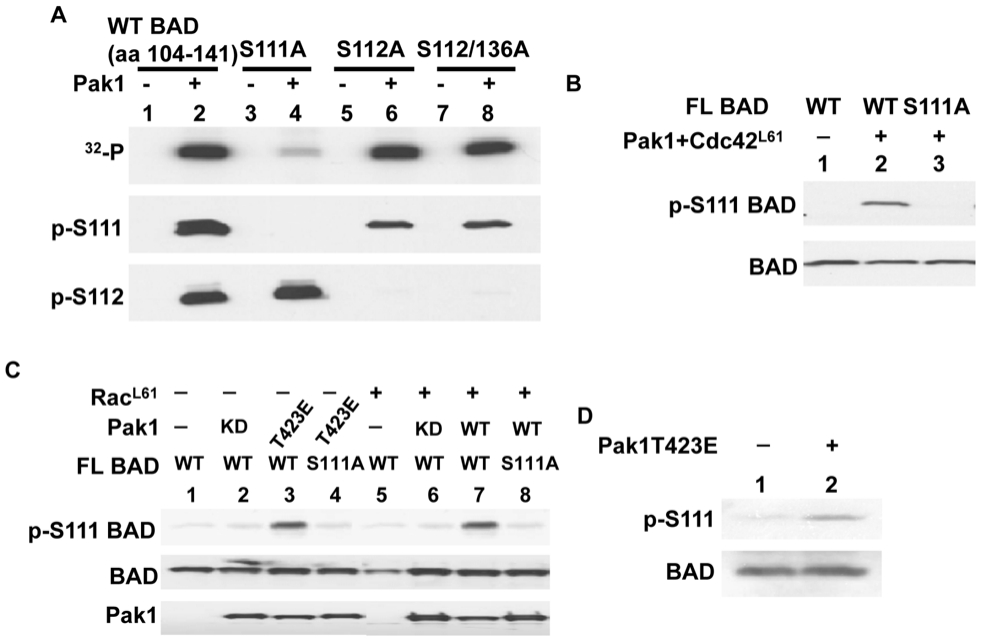
Phosphorylation of BAD at S111 by Pak1 in 293T cells. (A) Characterization of S111 and S112 antibodies used in this study. Purified GST-WT BAD (aa 104-141) and BAD (aa 104-141) mutants as indicated in the figure were incubated with or without Pak1 and phosphorylated with γ- [^32^P] ATP for 30 min at 30°C (upper panel) and subjected to autoradiography, or phosphorylated with unlabeled ATP (lower two panels) and probed with the S111 and S112 antibodies. (B and C) 293T cells were transfected with the indicated plasmids along with a BAD WT or S111A mutant plasmid. Pak1T423E, CDC42^L61^ and Rac^L61^ are activated mutants of Pak, Cdc42 and Rac respectively. Pak1 KD is a kinase dead Pak1 mutant. Equal amounts of extracts were incubated with glutathione beads in a GST pulldown experiment. Samples were separated by 10% SDS-PAGE and then blotted with anti-phospho-S111 BAD antibody to assess BAD phosphorylation. Total GST-BAD and Pak1 are shown in the lower panels. (D) Endogenous BAD was immunoprecipitated with an anti-BAD antibody and blotted with S111 phospho-specific antibody. Total BAD is shown below the blot.

### Phosphoamino Acid Analysis

After phosphorylation and electrophoresis, the phosphorylated BAD protein band was cut out from the gel, ground up and eluted with 50 mM NH_4_HCO_3_, 0.1% SDS and 0.5% β-mercaptoethanol twice in a combined volume of 1.2 mL. 20 µg of carrier protein (RNase A) and TCA (final concentration 20%) were added. The mixture was kept on ice for 1 hr and then spun down at 10,000 rpm for 10 min at 4°C. After washing with 95% ethanol and dry in air, the pellet was hydrolyzed in 50 µL 6 N HCl for 1 hr at 110°C, lyophilized and dissolved in 6 µL of pH 1.9 buffer (2.2% formic acid, 7.8% [V/V] glacial acetic acid) containing 60 µg/ml unlabeled phospho-amino acids. The sample was loaded onto a TLC plate (Selecto Scientific, GA). The TLC plate was run in a Multiphor 2-D apparatus (Amersham Pharmacia Biotech). The first dimension was run at 1.5 kV in pH 1.9 buffer for 20 min. After air-drying for 30–60 min, the TLC plate was turned 90° counter-clockwise and run at 1.4 kV in pH 3.5 buffer (0.5% glacial acetic acid, 0.05% of pyridine, V/V) for 16 min. After drying, the plate was sprayed with 0.25% (V/V) ninhydrin in acetone and then incubated at 65°C for 15 min to visualize the unlabeled phosphoamino acid markers which appear as purple spots identifying to the positions of p-Ser, p-Thr and p-Tyr. The plate was then exposed to X-ray film to identify the radiolabeled amino acids.

## Results

Schurmann et. al. demonstrated that Pak1 phosphorylated BAD on S112 and S136 but suggested that other sites might also be phosphorylated [24]. Since recent data suggested that Pak1 phosphorylated BAD indirectly through Raf-1 [15], we decided to re-examine if Pak1 could phosphorylate BAD directly. Kinase assays were performed using recombinant GST-BAD (aa 104-141) as a substrate for Pak1. ^32^P labeled ATP was readily incorporated into BAD when Pak1 was activated by Cdc42^L61^ (Figure 1A, lanes 1) but only a small amount of ^32^P-ATP was incorporated into BAD without Cdc42^L61^ (Figure 1A, lanes 6). To map the binding site, we mutated the two reported Pak1 phosphorylation sites, serines 112 and 136 to alanines. Mutants of either site and the S112/136A double mutant of BAD were all phosphorylated to a comparable extent (Figure 1A, lanes 2–4). Next, using 2D electrophoresis, we identified the phosphorylated amino acid as serine (Figure 1B); the serines within amino acids 104–141 are located at 108, 111, 128 and 134. To identify the serine residues that could be phosphorylated by Pak1, we constructed additional mutations in the S112/136A protein, and then purified and tested these GST fusion proteins in kinase assays (Figure 1C). Mutations in 108, 128 or 134 in combination with S112/136A did not reduce phosphorylation, but mutating S111 in combination with S112/136A abolished almost all phosphorylation on BAD (Figure 1C, lanes 2–6). Additional constructs that mutated S111 alone or in combination with S112/136A confirmed that only mutation in S111 significantly affected Pak1 phosphorylation of BAD (Figures 1D and 1E). Similarly, an *in vitro* kinase assay using BAD peptides (aa 105–118) showed that only mutation of S111 significantly reduced Pak1 and Rac^L61^ phosphorylation of BAD in this region (Figure S1D). The Coomassie brilliant blue staining showed equal loadings of proteins (Figures S1A and B). The position of phosphorylated Pak1, Cdc42^ L61^, and BAD WT and mutants were determined according to their sizes as shown in the Figure S1C.

Since S111 has not been reported as a Pak1 phosphorylation site, we examined if this site can be phosphorylated in cells. First, we designed and raised a phospho-specific antibody against S111 and characterized this antibody using *in vitro* kinase assays (Figure 2A). To do this, purified peptides (aa 104–141) of GST-wild type BAD, GST-BAD S111A, GST-BAD S112A, and GST-BAD S112/136A were incubated with or without Pak1 and ATP. The antibody against phospho-S111 recognized phosphorylated GST-wild type BAD, GST-BAD S112A, and GST-BAD S112/136A but not GST-BAD S111A (Figure 2A), indicating this antibody is specific in detecting phospho-S111 of BAD. As a control, we also tested a commercial antibody against phospho-S112 of BAD, which recognized S112 phosphorylated GST-wild type BAD and GST-BAD S111A but not the BAD peptides with mutated S112 (Figure 2A). When ^32^P-labeled ATP was used as a substrate, only the S111A mutant was a poor substrate as expected from the experiments in figure 1 (Figure 2A, upper panel lane 2). Coomassie brilliant blue staining of the purified proteins are shown in Figure S1C. Next, we determined whether Pak1 phosphorylates BAD at S111 in cells. In HEK293T cells, co-transfection of Pak1 with activated Cdc42 (Cdc42^ L61^, Figure 2B lane 2), activated Rac (Rac^L61^, Figure 2C lane 7), or an activated form of Pak1, Pak1T423E (Figure 2C lane 3), all stimulated BAD S111 phosphorylation to the same extent. No signal for BAD S111 phosphorylation was observed with a kinase dead Pak1 (KD or K299R, Figure 2C lane 2). Mutation of serine 111 to alanine (S111A) prevented Pak1 phosphorylation of S111 (Figure 2B lane 3, and Figure 2C lane 4 and 8). In cells transfected with Pak1T423E alone, the BAD phospho-S111 antibody was able to detect the endogenous phosphorylation of BAD at S111 (Figure 2D, lane2). These experiments demonstrated that Pak1 phosphorylated BAD at a unique site-S111 while we calculated from figure 1 that only 9.8% of the phosphate was directly incorporated into other serine sites, primarily at S112 (Figure 1D and E, and Figure 2A).

**Figure 3:**
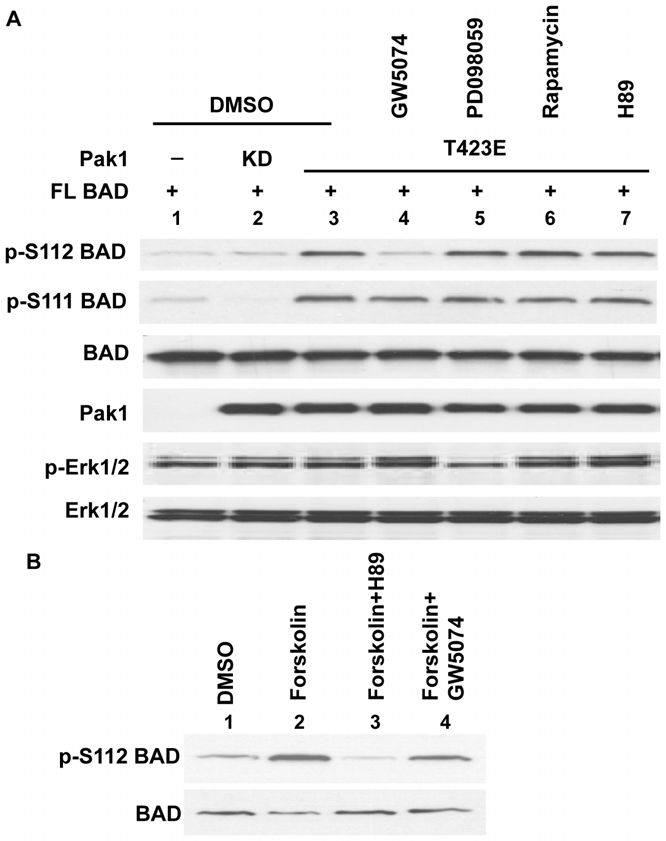
Pak1 stimulates phosphorylation of Ser112 on BAD via Raf-1. (A) Effects of kinase inhibitors on BAD S112 phosphorylation. 293T cells were co-transfected with expression vectors encoding full length (FL) WT BAD and Pak1 (KD or T423E). 24 hr after transfection, cells were starved for 16 hr and treated with GW5074 (5 µM), PD098059 (20 µM), Rapamycin (5 µM) or H89 (5 µM) for 2.5 hr as indicated. Equal amounts of proteins were used for Western blot to assess BAD phosphorylation at S112 or S111. The cell lysates were also subjected to immunoblotting with anti-BAD, anti-Myc, anti-phospho-ERK and anti-ERK antibodies. (B) Effects of inhibitors on forskolin stimulated activation of BAD phosphorylation. 293T cells were transfected with full length (FL) WT BAD for 24 hr, starved for 16 hr and treated with vehicle (DMSO), GW5074 (5 µM), or H89 (5 µM) for 2.5 hr prior to forskolin (50 µM) treatment. Equal amounts of proteins were used for Western blot to assess BAD phosphorylation at S112. The cell lysates were also subjected to immunoblotting with anti-BAD.

Expression of Pak1T423E, but not kinase dead Pak1 (KD), stimulates phosphorylation of BAD at S112 (Figure 3A lane 2 and 3) [23]–[26]. However, as shown in figure 1, Pak1 phosphorylation at S112 is likely to be indirect since only a small fraction of phosphate is incorporated into BAD at serine 112. Since S112 of BAD was such a poor phosphate acceptor for Pak1, we hypothesized that other protein kinases downstream of Pak1 phosphorylate BAD at S112. Cells were co-transfected with pEBG-WT BAD (full length) and empty vector, KD Pak1 or Pak1T423E, and then treated with the Raf-1 inhibitor GW5074 (5-Iodo-3-[(3,5-dibromo-4-hydroxyphenyl)methylene]-2-indolinone), a MEK inhibitor (PD098059), a p70S6K inhibitor (rapamycin), or a PKA inhibitor (H89). Pak1 stimulation of S112 phosphorylation was not inhibited by PD098059, rapamycin, or H89 (Figure 3A lane 5–7). However, the Raf-1 inhibitor GW5074 reduced phosphorylation of BAD at S112 to the control levels (Figure 3, lane 4). This confirms earlier studies with the less specific Raf inhibitor BAY 43-9006 (Sorafenib) suggesting that Raf-1 acts downstream of Pak1 to phosphorylate S112 of BAD [15]. We also assessed the phosphorylation of ERK, a downstream target of the Raf-1 cascade. Activated Pak1 did not obviously stimulate ERK phosphorylation (Figure 3A lane 3). Phosphorylation of ERK was slightly induced by the GW5074 but reduced by PD098059 (Figure 3A lane 4 and 5). Importantly, the Raf-1 inhibitor prevented Pak1-dependent phosphorylation of S112 but not phosphorylation at S111 (Figure 3A lane 4). Similar effects by Raf-1 on phosphorylation of S111 and S112 were observed when dominant negative mutants of Raf-1 were used to inhibit Raf-1 (data not shown). To exclude the possibility that the Raf-1 inhibitor interfered with the PKA pathway, we tested the effect of the Raf-1 inhibitor on PKA-induced S112 phosphorylation. 293T cells were transfected with pEBG-WT BAD for 24 hr, starved for 16 hr and pretreated with vehicle (DMSO), the PKA inhibitor H89 or the Raf-1 inhibitor GW5074 for 30 min and then stimulated with forskolin to activate adenylyl cyclase. H89 inhibited forskolin stimulated S112 phosphorylation, as expected, but the Raf-1 inhibitor had little effect (Figure 3B). An *in vitro* assay also showed that a mutation at S111 does not influence PKA phosphorylation of BAD at S112 (Figure S2A). These data demonstrate that, unlike the case with Pak1, PKA does not stimulate S112 phosphorylation through Raf-1 and most likely phosphorylates BAD directly at S112.

**Figure 4:**
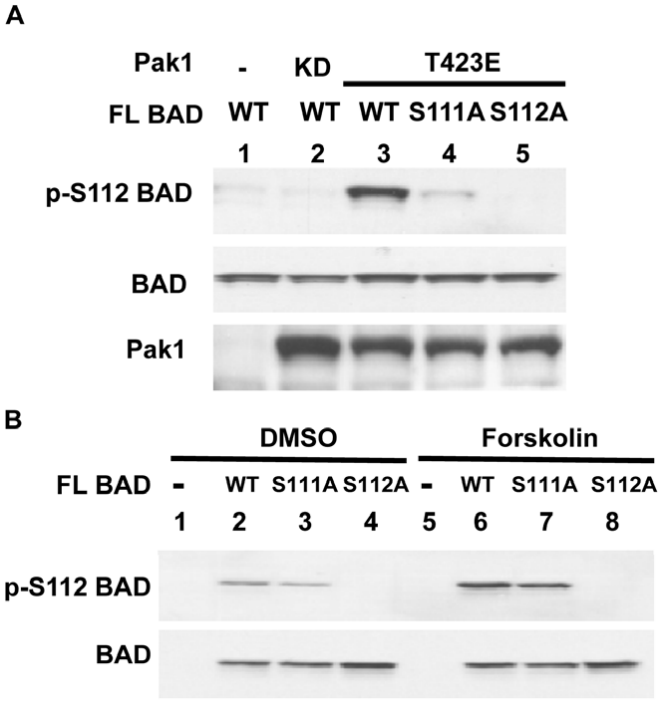
Mutations on BAD S111 reduces Pak1 phosphorylation of BAD at S112. (A) 293T cells were co-transfected with expression vectors encoding full length (FL) BAD (WT, S111A, or S112A) and Pak1 (KD or T423E). Equal amounts of proteins were used for Western blotting to assess BAD phosphorylation at S112. The cell lysates were also subjected to immunoblotting with anti-BAD and anti-Pak1 antibodies. (B) 293T cells were transfected with expression vectors encoding full length (FL) BAD (WT, S111A, or S112A). Equal amounts of proteins were used for Western blot to assess BAD phosphorylation at S112. The cell lysates were also subjected to immunoblotting with anti-Pak1 antibodies.

To determine if preventing phosphorylation at S111 would influence S112 phosphorylation, we tested if a mutation of S111 would affect phosphorylation at S112. Again, 293T cells were co-transfected with expression vectors encoding full length (FL) BAD (WT, S111A, or S112A) and Pak1 (KD or T423E). We found that mutating S111 to alanine in full length BAD reduced Pak1 stimulated phosphorylation at S112 (Figure 4A lane 4). However, there was no effect on forskolin stimulated (PKA dependent) phosphorylation of S112 (Figure 4B lane 7). Thus Pak1 phosphorylation of S111 enhances S112 phosphorylation by Raf-1. Using BAD peptides (aa 105–118), we found that activated Raf-1 enhanced Pak1 phosphorylation of BAD (Figure S2B Lane 1 and 2). Activated Raf-1 alone, however, did not phosphorylate phosphorylated S111 (pS111) or S111A (data not shown). Moreover, activated Pak1 alone did not phosphorylate phosphorylated S111 (pS111) or S111A but addition of activated Raf-1 led to a weak phosphorylation of pS111 and S111A (Figure S2B Lane 3–6). Incubating Pak1 with or without Raf-1 failed to phosphorylate pS112 or S111/112A (Figure S2B Lane 7–10). These data indicate that modification of S111 *in vitro* may affect the efficient S112 phosphorylation by Raf-1 and Pak1. Furthermore, these data showed that pS112 reduces phosphorylation of S111 by Raf-1 or Pak1. Together, these studies demonstrate that Pak1 phosphorylates BAD directly on S111 *in vivo* and indirectly on S112 through Raf-1.

**Figure 5:**
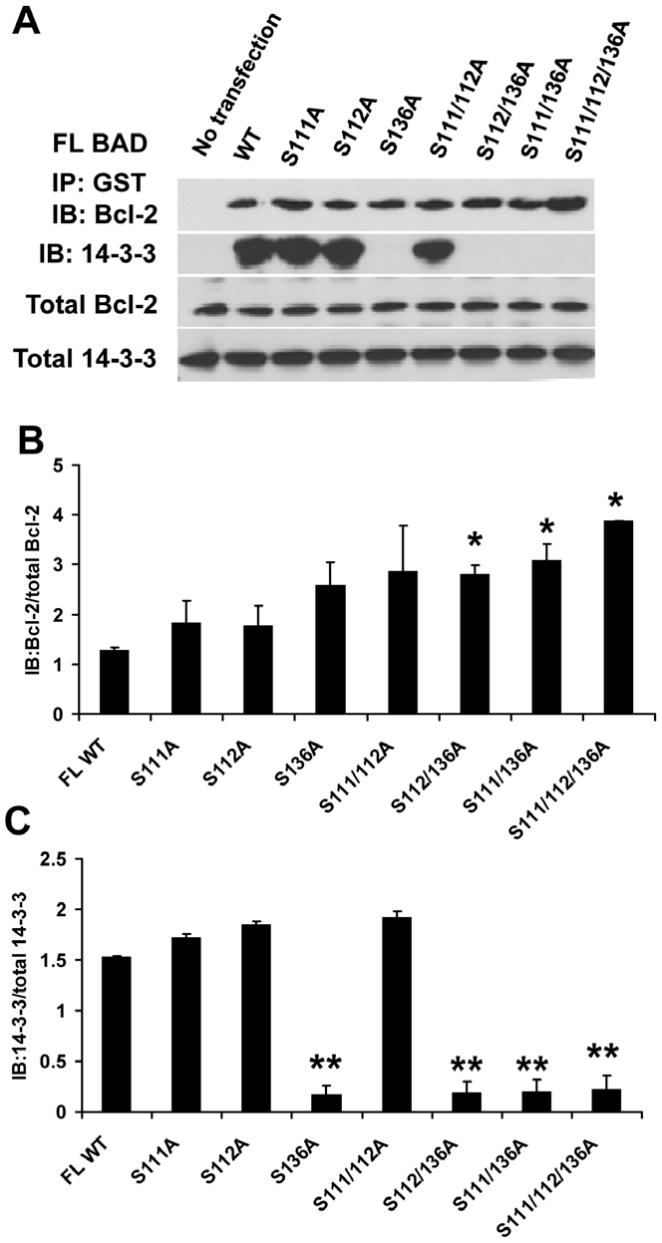
Bcl2 and 14-3-3 binding to BAD and model. (A) Effects of S111, S112 and S136 mutation(s) on BAD/Bcl-2 and BAD/14-3-3 complex formation. 293T cells were transfected with the full length (FL) BAD WT or mutant plasmids as indicated in the figure. Equal amounts of extracts were incubated with glutathione beads in a GST pulldown experiment. Samples were separated by 10% SDS-PAGE and then blotted with anti-Bcl-2 or anti 14-3-3 antibodies. Total Bcl-2 and 14-3-3 were also determined by Western blotting. (B and C) Protein levels were evaluated through densitometry and two different blots for were quantified. The ratio of immunoprecipitated and blotted Bcl-2 to total Bcl-2, and immunoprecipitated and blotted 14-3-3 to total 14-3-3 were plotted as B and C.

Phosphorylation of BAD at S112 and S136 decreases BAD/Bcl-2 complex formation and increases BAD/14-3-3 binding, which results in increased cell survival [2]. To determine if phosphorylation of BAD at S111 affects complex formation, GST pull down assays were performed with extracts from cells transfected with pEBG-WT BAD (full length) or different mutants as indicated in figure 5. Mutation of S111, S112, S136, or combinations of double mutations caused a slight increase of Bcl-2/BAD binding (Figure 5A and B). A triple mutation of the S111, S112 and S136 sites led to the most Bcl-2/BAD complex formation (Figure 5A lane 9 and B). We also tested 14-3-3 binding to BAD and found that binding was dramatically decreased in mutants with S136A mutations (Figure 5A and C), indicating that S136 is the most important phosphorylation site for the BAD/14-3-3 complex formation as previously observed [32], [33]. The enhanced Bcl-2 binding by the triple mutant is likely the result of the release of BAD from 14-3-3 sequestering in addition to the enhanced Bcl-2 association.

**Figure 6:**
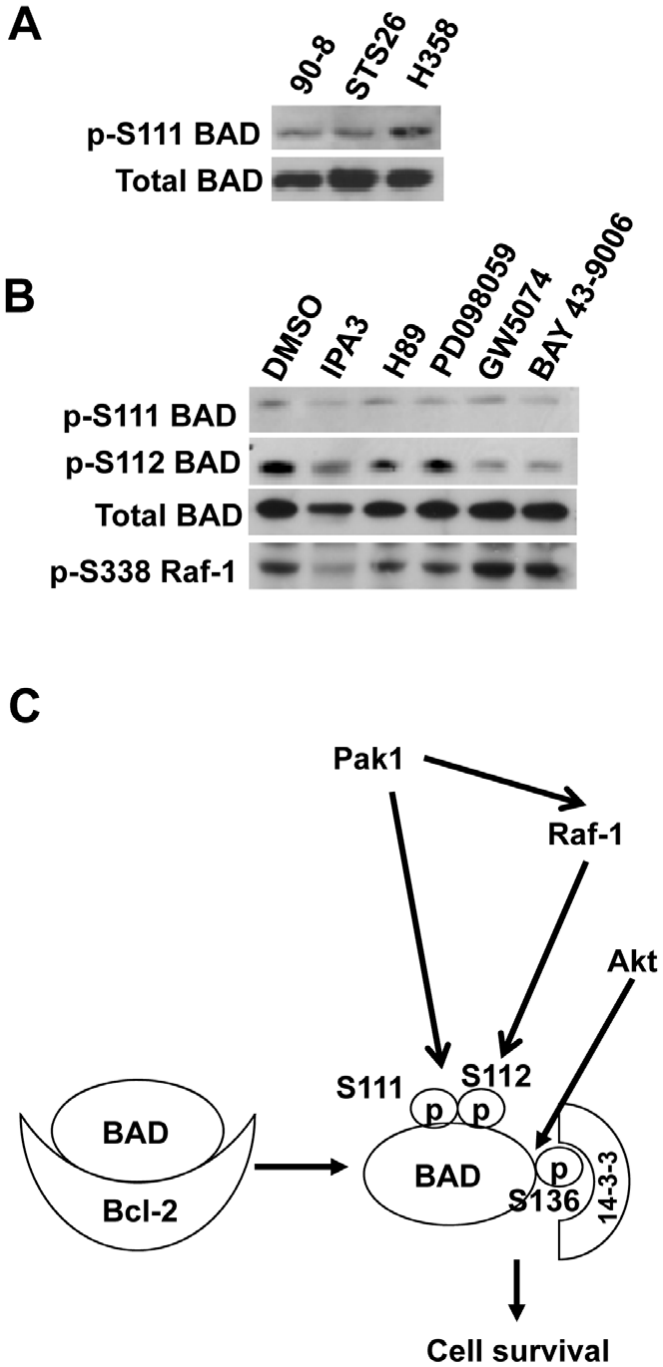
Pak1 inhibition reduced BAD S111 and S112 phosphorylation. (A) BAD S111 phosphorylation was observed in a lung cancer cell line H358 and the MPNST cell lines 90-8 and STS26. (B) Effects of protein kinase inhibitors on BAD S111 and S112 phosphorylation. The MPNST cells, ST88-14, were starved and treated with vehicle (DMSO), IPA3 (20 µM), GW5074 (5 µM), or H89 (5 µM) for 15 hr. Equal amounts of proteins were used for Western blot to assess BAD phosphorylation at S111and S112. The cell lysates were also subjected to immunoblotting with anti-BAD. As a control, Raf-1 phosphorylation at S338 was examined. (C) Model showing that Pak1 phosphorylates BAD at S111 directly, but most of its protective effects are mediated through Raf-1 and phosphorylation of S112. S111 also appears to facilitate phosphorylation at S112 because mutating it reduces phosphorylation by Pak1, but not phosphorylation by PKA. S136 is the most important phosphorylation site for the BAD/14-3-3 complex formation as previously observed. The enhanced Bcl-2 binding by the triple mutant is likely the result of the release of BAD from 14-3-3 sequestering in addition to the enhanced Bcl-2 association.

To address if S111 of BAD is phosphorylated in other cell lines besides 293T cells, we probed a panel of malignant peripheral nerve sheath tumor (MPNST) cells and one lung cancer cell line (H358) (Figure 6). The MPNST cell lines included ST88-14, STS26, and 90-8 as well as a rat Schwannoma RT-4. We found that Bad was phosphorylated on S111 in all of the cell lines except RT-4, which expressed very low levels of BAD (Figure 6A, and B lane 1; data not shown for RT-4 cells). We chose to use ST88-14 cells to examine the effects of Pak1 inhibition on BAD phosphorylation since Pak regulates ERK in them [34]. Upon treatment with 20 µM IPA-3, a Pak1, Pak2 and Pak3 inhibitor [35], ST88-14 cells underwent apoptosis (data not shown). When we treated ST88-14 with various kinase inhibitors (Figure 6B lane 3–6), we found that the Pak inhibition reduced phosphorylation of Bad at S111 and S112. The Raf inhibitors only reduced phosphorylation at S112 but not S111. PD098059 did not have any effect on either site, while H89 had a partial effect on S112, but not S111. IPA-3 also reduced phosphorylation of Raf-1 at S338, confirming that Raf-1 is a downstream target of Pak1. We noted that exposure to IPA-3 reduced the overall levels of BAD, which appears to account for most of the effects on S111 phosphorylation, but not the much stronger loss of phosphorylation seen at S112. The reduction of BAD upon treatment with IPA-3 was more pronounced upon longer exposure to IPA-3 suggesting that Pak1 may also affect the stability of BAD (data not shown).

## Discussion

There are several reports of Pak1 stimulating BAD phosphorylation at both S136 and S112 [15], [16], [23], [24]. While most studies relied on cell based assays, some documented the phosphorylation *in vitro*, usually using phosphospecific antibodies [16], [24], [36]. We showed here, with radioisotope, a more quantitative assay system, that there was still a substantial phosphorylation of BAD with the S112 and S136 double mutant. This was because the phosphorylation of BAD occurs primarily on amino acid S111. Studies with peptides have found that the preferred site of phosphorylation for Pak1 are peptides with basic amino acids R and K at positions -2 and -3 from the phosphorylated serine and a hydrophobic residue such as tyrosine at the +2 position. The sequence of BAD at the region phosphorylated by Pak1 is ETRSRHSSYPAGTEE (SS are serines 111 and 112, respectively). With R at position -2 and Y at position +2, S111 fits well with the preferred motif although it lacks a basic residue at -3. S112, however, has only a single preferred component of the preferred motif, R at position -3. In comparison to other Pak1 substrates, S111 is very similar to two other widely documented sequences that Pak1 phosphorylates, S338 of Raf-1, RPRGQRD**S**SYYWEIE and S297 of MEK, RTPGRPL**S**SYGMDSR. All have in common a single upstream basic amino acid, phosphorylation at the first of two serines (Raf is additionally phosphorylated, but to a lesser extent, at the second serine), and a tyrosine at the +2 position. We note, however, that Pak1 substrates are difficult to predict from sequence comparisons. For example, we did not find BAD, Raf-1 or MEK listed among 1433 possible targets identified in a Scansite search of the Swiss protein database [37] using a sequence matrix developed by Peterson's lab [38] (data not shown).

While there are numerous reports documenting phosphorylation at S112 *in vivo* and *in vitro*, S111 has only recently been found to be phosphorylated. However, both reports were large scale proteomics studies of phospho-proteins [39] and the responsible kinases were not identified. Here, for the first time, we document Pak1 as a kinase that phosphorylates BAD at S111 although the experiments with the Pak inhibitor suggest that other kinases also phosphorylate this site. We propose that while Pak1 phosphorylates BAD at serine 111, most of Pak1 protective effects are through Raf-1 mediated phosphorylation of BAD at serine 112 (Figure 6C). Serine 111 appears to facilitate phosphorylation at S112 because mutating it reduces phosphorylation by Pak1, but not PKA (Figure 4). Raf-1 is widely studied for its role in cell proliferation through phosphorylation of MEK and ERK. Increasingly, MEK independent signals have been identified to promote cell survival. The MEK independent survival signals play a major role in the physiology of B-Raf and Raf-1. Cells from knockouts of both B-Raf and Raf-1 display normal proliferative rates but increased rates of apoptosis [14], [40]–[44] while knockouts of MEK1 display no increases in apoptosis [45]. Cells from Raf-1 knockouts also have wild type levels of ERK activation, suggesting that the survival signals are independent of MEK and ERK. Our data suggest Pak1 and Raf-1 cooperate to maintain BAD phosphorylation at S112 but not at S111, and that MEK signaling is not involved in phosphorylation at either site.

An unexpected observation from this study is that there is a difference in the sensitivity of downstream pathways to Raf inhibitors. We found that a Raf-1 inhibitor reduced phosphorylation of BAD at S112 in 293T cells under a condition where it actually slightly increased phosphorylation of Erk1 (Figure 3A, lane 4). This difference may be caused by different isoforms of Raf signaling to the two pathways. For example, in some cells, B-Raf may be the primary kinase for the ERK pathway while Raf-1 may be the primary kinase for BAD. It was recently shown that low doses Raf inhibitors stimulated ERK in cells that harbor wild-type Raf and/or activated Ras. This is because inactivated B-Raf forms heterodimers with Raf-1, which then becomes activated to stimulate ERK [46]–[49]. Our studies raise the possibility that Raf inhibitor-activated Rafs may be unable to phosphorylate BAD at S112.

The role of Raf in cell survival pathways may explain why Raf inhibitors are performing better in the clinic than MEK inhibitors [46]. Since MEK inhibitors do not prevent BAD phosphorylation, they only partially disrupt Raf signaling. We speculate that blocking the survival signals may be required for clinical responses to Raf inhibitors. The survival signals appear to be especially important for liver cells since liver carcinomas respond well to Raf inhibitors, such as Sorafenib, and Raf-1 knockout mice have high rates of liver apoptosis [14], [42]. Our studies support the expanding role of Pak1 in cell survival signals through Raf-1 regulation of BAD phosphorylation and suggest that Pak1 inhibitors may enhance the effectiveness of MEK inhibitors by reducing MEK-independent signals from Raf (Figure 6C). The observation of BAD S111 phosphorylation in a lung cancer cell line and several MPNST cell lines suggests that S111 phosphorylation could be used as pharmacodynamic marker of Pak activity in some cancers.

## Supporting Information

Figure S1 ***In vitro*** **protein and peptide kinase assays.** (A) Coomassie brilliant blue staining of the gel of Figure 1C. (B) Coomassie brilliant blue staining of the gel of Figure 1D. (C) Coomassie brilliant blue staining of the purified proteins. All proteins were purified from *E. coli* through glutathione sephorase 4B columns except His-Pak1 which was purified from sf 9 insect cells through a Ni-NTA agarose column. Wild type (WT) and mutant GST-BAD fusion proteins contain a murine BAD fragment (aa 104-141). 2 μg of His-Pak1, 1μg of GST-Cdc42^L61^, 2 μg of GST and 6 μg of GST-BAD proteins were applied to 10% SDS-PAGE and stained with Coomassie Brilliant Blue. (D) Purified BAD peptides (aa 105-118) were incubated with Rac^L61^, Pak1 or both with γ^32^P-ATP and subjected to autoradiography.
(TIF)

Figure S2 (A) Purified GST-BAD was incubated with or without PKA and phosphorylated with γ^32^P-ATP ATP (upper panel) and subjected to autoradiography, or phosphorylated with unlabeled ATP (lower two panels) and probed with the S111 and S112 antibodies. (B) Purified BAD peptides (aa 105-118) were incubated with activated Raf-1, Pak1 or both with γ^32^P-ATP ATP, run on a tricine gel and subjected to autoradiography. Bottom panel of B shows phosphorimager quantification of the bands in panel D (n = 2). Student t-test was performed and * p<0.05. The arrows indicate the bands that were quantified.
(TIF)
