# Supplementary figures and images for: p21-Activated Kinase 1 (Pak1) Phosphorylates BAD Directly at Serine 111 In Vitro and Indirectly through Raf-1 at Serine 112
Source: PLoS One. 2011 Nov 11;6(11):e27637. doi: 10.1371/journal.pone.0027637 (PMC3214075; doi:10.1371/journal.pone.0027637)

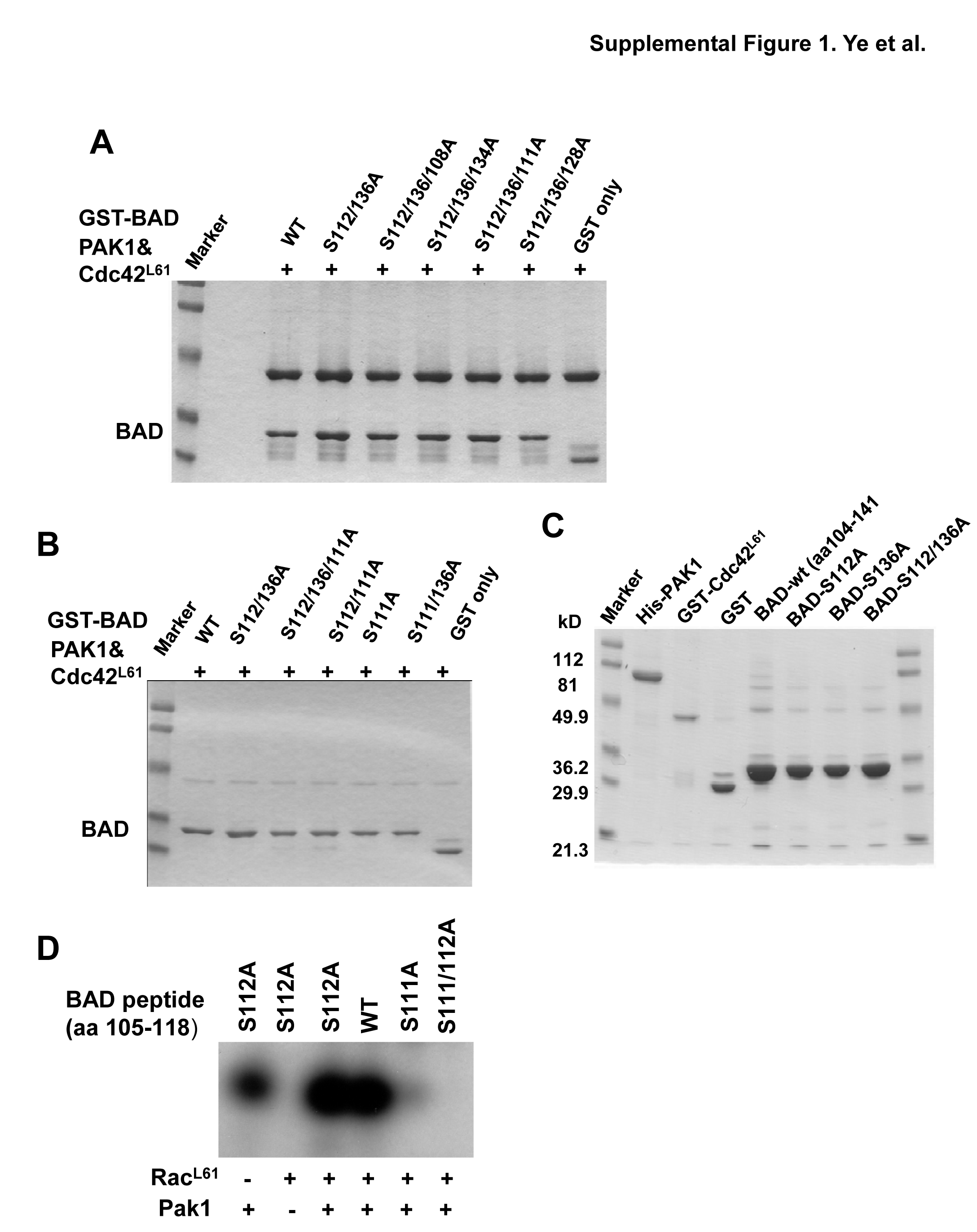

Supplement: Figure S1 — In vitro protein and peptide kinase assays. (A) Coomassie brilliant blue staining of the gel of Figure 1C. (B) Coomassie brilliant blue staining of the gel of Figure 1D. (C) Coomassie brilliant blue staining of the purified proteins. All proteins were purified from E. coli through glutathione sephorase 4B columns except His-Pak1 which was purified from sf 9 insect cells through a Ni-NTA agarose column. Wild type (WT) and mutant GST-BAD fusion proteins contain a murine BAD fragment (aa 104-141). 2 μg of His-Pak1, 1μg of GST-Cdc42L61, 2 μg of GST and 6 μg of GST-BAD proteins were applied to 10% SDS-PAGE and stained with Coomassie Brilliant Blue. (D) Purified BAD peptides (aa 105-118) were incubated with RacL61, Pak1 or both with γ32P-ATP and subjected to autoradiography. (TIF) [file pone.0027637.s001.tif]

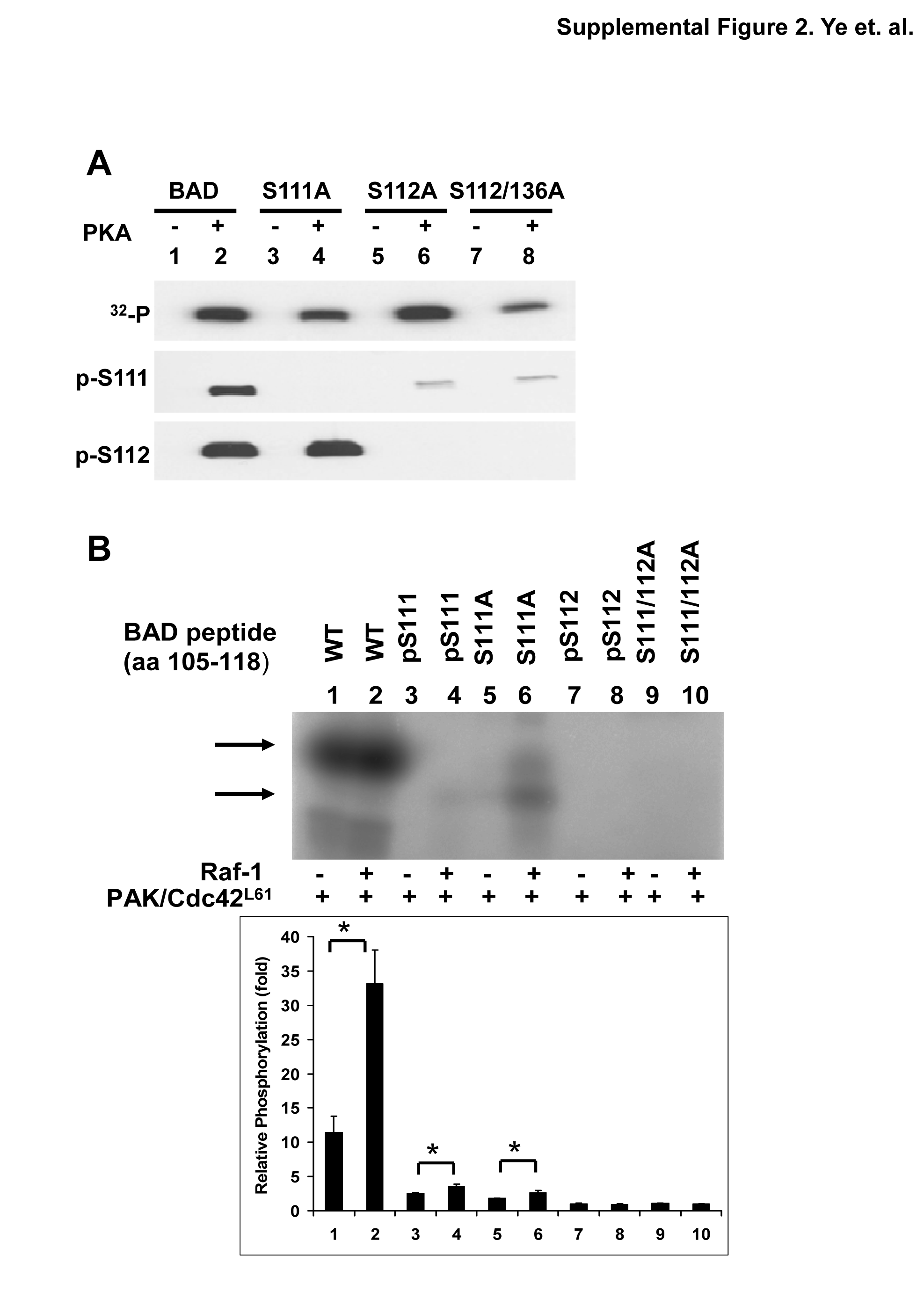

Supplement: Figure S2 — (A) Purified GST-BAD was incubated with or without PKA and phosphorylated with γ32P-ATP ATP (upper panel) and subjected to autoradiography, or phosphorylated with unlabeled ATP (lower two panels) and probed with the S111 and S112 antibodies. (B) Purified BAD peptides (aa 105-118) were incubated with activated Raf-1, Pak1 or both with γ32P-ATP ATP, run on a tricine gel and subjected to autoradiography. Bottom panel of B shows phosphorimager quantification of the bands in panel D (n = 2). Student t-test was performed and * p<0.05. The arrows indicate the bands that were quantified. (TIF) [file pone.0027637.s002.tif]
